# Meprin β knockout reduces brain Aβ levels and rescues learning and memory impairments in the APP/lon mouse model for Alzheimer’s disease

**DOI:** 10.1007/s00018-022-04205-5

**Published:** 2022-03-02

**Authors:** Liana Marengo, Fred Armbrust, Caroline Schoenherr, Steffen E. Storck, Ulrich Schmitt, Silvia Zampar, Oliver Wirths, Hermann Altmeppen, Markus Glatzel, Christoph Kaether, Sascha Weggen, Christoph Becker-Pauly, Claus U. Pietrzik

**Affiliations:** 1 Institute for Pathobiochemistry, University Medical Center of the Johannes Gutenberg University Mainz, Mainz, Germany; 2 Institute of Biochemistry, Unit for Degradomics of the Protease Web, Christian-Albrechts-University Kiel, Kiel, Germany; 3 Leibniz-Institute for Resilience Research, Mainz, Germany; 4 Department of Psychiatry and Psychotherapy, University Medical Center Göttingen (UMG), Göttingen, Germany; 5 Institute of Neuropathology, University Medical Center HH-Eppendorf, Hamburg, Germany; 6 Leibniz Institute of Aging-Fritz Lipmann Institute, Jena, Germany; 7 Department of Neuropathology, Heinrich Heine University, Düsseldorf, Germany; 8 Molecular Neurodegeneration, Institute for Pathobiochemistry, University Medical Center of the Johannes Gutenberg University of Mainz, Duesbergweg 6, 55099 Mainz, Germany

**Keywords:** Meprin β, Alzheimer’s disease, Amyloid precursor protein, Truncated Aβ, APP(V717I)

## Abstract

β-Site amyloid precursor protein (APP) cleaving enzyme-1 (BACE1) is the major described β-secretase to generate Aβ peptides in Alzheimer’s disease (AD). However, all therapeutic attempts to block BACE1 activity and to improve AD symptoms have so far failed. A potential candidate for alternative Aβ peptides generation is the metalloproteinase meprin β, which cleaves APP predominantly at alanine in p2 and in this study we can detect an increased meprin β expression in AD brain. Here, we report the generation of the transgenic APP/lon mouse model of AD lacking the functional *Mep1b* gene (APP/lon × *Mep1b*^−/−^). We examined levels of canonical and truncated Aβ species using urea-SDS-PAGE, ELISA and immunohistochemistry in brains of APP/lon mouse × *Mep1b*^−/−^. Additionally, we investigated the cognitive abilities of these mice during the Morris water maze task. Aβ1-40 and 1–42 levels are reduced in APP/lon mice when meprin β is absent. Immunohistochemical staining of mouse brain sections revealed that N-terminally truncated Aβ2–*x* peptide deposition is decreased in APP/lon × *Mep1b*^−/−^ mice. Importantly, loss of meprin β improved cognitive abilities and rescued learning behavior impairments in APP/lon mice. These observations indicate an important role of meprin β within the amyloidogenic pathway and Aβ production in vivo*.*

**Supplementary Information:**

The online version contains supplementary material available at 10.1007/s00018-022-04205-5.

## Introduction

The β-site APP cleaving enzyme 1 (BACE1) has been identified as a membrane bound aspartyl protease and it is considered the main β-secretase to generate amyloid β peptides (Aβ) in neurons. This became particularly evident when BACE1 knockout (BACE1^−/−^) mice showed a dramatic decrease in Aβ production once crossed to APP-overexpressing transgenic mouse lines such as Tg2576 [1] or to 5× FAD mice [2]. This decrease was accompanied by improvements in Alzheimer’s disease (AD)-related cognitive deficits, such as memory and learning impairment [3]. Based on this observation, several BACE1 inhibitors were developed, reaching clinical trials as potential new treatments for AD [4]. Strikingly, many of those therapeutic strategies have been suspended due to their failure to improve AD symptoms [5]. Furthermore, recent evidence indicated that complete blockage of BACE1 can lead to considerable side-effects, e.g., interfering with neural progenitor cell proliferation, resulting in a decreased number of neurons [6] and synaptic plasticity impairment [7]. These effects can be explained by observations from analysis of BACE1^−/−^ mice, such as elevated pain sensitivity, reduced grip strength [8] and epileptic seizures [9]. BACE1 depletion leads to accumulation of unprocessed type 3 neuregulin 1 (NRG1) and to hypomyelination in the hippocampus and peripheral nerves [10]. Moreover, BACE1 inhibition increases neuronal cell surface levels of seizure protein 6 (Sez6), which controls synaptic connectivity and motor coordination in mice [11]. Due to its importance in the brain, these data suggest that BACE1 might not be the best therapeutic target since its chronic or acute pharmacological manipulation would give rise to many deleterious consequences.

Aβ peptides are generated through the proteolytic processing of the Amyloid Precursor Protein (APP) by the sequential action of β- and γ-secretase. Apart from the canonical ‘full-length’ Aβ (1–40, 1–42), multiple Aβ species bearing different N and C-termini have been described [12–14] The exact physiological role of the N-terminally truncated forms of Aβ (Aβ*x*–40/ *x*–42) is still debated, but they are detected as major components in extracellular amyloid deposits in human brain. In fact, N-terminal truncations accounted for more than 70% of total Aβ species detected in human brain tissues from patients diagnosed with severe AD [15, 16], thus emphasizing their important role in the pathology of the disease (reviewed in [17]). Since BACE1 can only generate Aβ peptides starting at position 1 or 11 (Aβ1–*x*/11–*x*), Aβ species starting at other N-terminal positions, e.g., 4 (Aβ4–x) or 2 (Aβ2–*x*), cannot be assigned to BACE1 activity [16, 18]. BACE1 knockout neurons do not secrete Aβ1–40/42 and Aβ11–40/42 [1] while N-terminally truncated Aβ peptides can still be detected under these conditions [19]. The existence of different Aβ species in the absence of BACE1 indicates an independent pathway of APP processing by alternative β-secretases. Over the last few years other enzymes, such as ADAMTS4 [20], Aminopeptidase A [21] and cathepsin B [22] have been described to be involved in BACE1–independent N-terminal truncated Aβ generation.

Another potential candidate for alternative Aβ production is the metalloproteinase meprin β. Meprin β was recently identified as a potential risk gene associated with AD through exome-wide association analysis in a cohort of patients clinically diagnosed with AD [23]. Fittingly, meprin β mRNA levels were shown to be increased in brains of AD patients compared to age-matched controls [24, 25]. Meprin β is predominantly membrane-bound and co-localizes with APP in the late secretory pathway or at the cell membrane [26]. Of note, it was demonstrated that meprin β cleaves APP and it is capable of liberating not only Aβ1–*x* , but also N-truncated Aβ peptides starting mainly at position 2 and 3 [24, 27], indicating a possible role of meprin β within the amyloidogenic pathway in addition to BACE1. Moreover, we have shown that meprin β even has a higher affinity to wild-type APP compared to BACE1 [24] and that the various familiar APP mutations differently affect meprin β cleavage specificity. The protective A673T APP mutant, which reduces BACE1 cleavage, also induced a 70% decrease in the Aβ2-40 generated by meprin β in vitro [26]. Importantly, the change in amino acid composition around the β-site in the ‘Swedish’ APP mutation (K670N/M671L) almost fully abolished generation of N-terminally truncated Aβ2-40/42 variants [26]. However, this Swedish mutation (APPswe) is commonly introduced into mouse models of AD because it strongly enhances overall Aβ production by BACE1, thus facilitating studies focusing on the role of Aβ in AD. As a consequence, though these transgenic mouse models have substantially broadened our knowledge on the alterations underlying AD pathology, they might have also supported to oversight certain other potentially relevant pathophysiological mechanisms. In this respect, the strong affinity of BACE1 for APPswe might have concealed effects from other important enzymes such as meprin β that could be mechanistically involved in AD pathogenesis. Based on increasing evidence for alternative β-secretases, we aimed at analyzing meprin β-dependent Aβ generation in vivo using an animal model for AD that does not harbor the Swedish mutation on the APP transgene. We now demonstrate that soluble Aβ levels are decreased in the brain of the AD-mouse model APP/lon mice when meprin β is absent. More specifically, we found a decrease in the deposition of the N-terminally truncated Aβ2–*x* in the cortex of these animals. Furthermore, we show that loss of meprin β improved cognitive impairments such as memory and learning.

## Materials and methods

### Human brain tissue

All post mortem brain samples of frontal isocortex from neuropathologically confirmed AD patients (*n* = 21, 10 females, 11 males; mean age ± SD: 77.9±10.1 years) and non-demented control patients (*n* = 17, 8 females, 9 males; 75.8 ± 11.6 years) were obtained from *University Medical Center HH-Eppendorf (UKE)*. AD cases were diagnosed as CERAD B-C, Braak stages III–VI. None of the controls suffered from dementia or any other neurodegenerative disease.

### Histological and immunohistochemical analysis of formalin-fixed paraffin embedded (FFPE) human brains

Sections of FFPE frontal isocortex from healthy control and AD were stained with a Bielschowsky staining kit according to standard laboratory procedures. Immunostainings of Aβ (using monoclonal antibody 6E10; 1:100, #39320, Biolegend, CA, USA), meprin β (using rabbit polyclonal anti-meprin β antibody; 1:500) and phosphorylated Tau protein (using monoclonal antibody AT8 (MN1020); 1:5000; Thermo Scientific) were performed with a Ventana Benchmark XT system according to common protocols. Meprin β-positive neurons were counted in three representative microscopic fields of approximately 1.87 mm^2^ (taken along cortical layers III to VI) per patient. The mean value of meprin β-positive cells derived from these three pictures per patient (corresponding to *n* = 1) was divided by the field area analysed in each experimental group and used for statistical analysis.

### Human brain lysates

Brain tissue of human frontal isocortex (3 controls and 3 AD) were lysed in RIPA buffer [50 mM Tris–HCl, pH 8.0, 150 mM NaCl, 1% Octylphenoxy polyethoxyethanol (IGEPAL), 0.1% Sodium dodecyl sulfate (SDS), 0.5% deoxycholate, 10 mM NaF, protease and phosphatase inhibitor cocktails] by homogenization in a glass homogenizer. Homogenates were incubated on ice for 30 min and centrifuged at 120,000×*g* at 4 °C for 30 min (TLA120.2 rotor, Beckman Coulter ultracentrifuge).

### Animals

hAPP[V717I] (APP/lon) [28], BACE1^−/−^ [29], meprin β knockout (*Mep1b*^−/−^) [30] and wild-type (WT) mouse strains were maintained on a 12 h light/dark cycle with food and water ad libitum. APP/lon mice lacking *Mep1b* were generated by crossing *Mep1b*^−/−^ mice into hAPP[V717I] mice, which we will refer to as APP/lon × *Mep1b*^−/−^.

### Mouse brain lysates

Animals were sacrificed by cervical dislocation and brains were removed. For the meprin β characterization, brains were dissected into cortex and hippocampus. For each animal group, we pooled the specific brain regions from five mice and homogenized in homogenization buffer (320 mM Sucrose, 10 mM HEPES, 1 mM EDTA) on a 1:6 ratio (weight:volume). Homogenates were centrifuged at 18,000×*g* for 30 min, the soluble extract was removed and the pellet was re-supended in 1% NP-40 lysis buffer (150 mM NaCl, 50 mM Tris–OH pH 8.0). Lysates were centrifuged at 18,000×*g* for 30 min and supernatants were collected for further analysis. For Aβ analysis, snap frozen hemispheres were mechanically homogenized in 0.01M PBS on the same above mentioned ratio and subsequently ultracentrifuged at 120,000×*g* for 30 min (TLA120.2 rotor, Beckman Coulter). The PBS-extracted supernatant (soluble fraction) was collected and the pellet was re-suspended in 800 µL of 0.01M PBS containing 2% SDS. Homogenates were ultracentrifuged at 120,000×*g* for 30 min and the SDS-extracted supernatant (insoluble fraction) was kept for further analysis. PBS and SDS protein extracts were stored at – 80 °C until use. All buffers were supplemented with protease (cOmplete, Roche) and phosphatase inhibitor cocktails (PhosSTOP, Roche).

### Immunoprecipitation of amyloid β

Soluble fractions from mouse protein extracts were used for immunoprecipitation (IP). Magnetic Dynabeads (M-280 Sheep Anti-Mouse IgG, 11201D, Invitrogen) containing sheep anti-mouse IgG attached to their surface were activated with the monoclonal antibody (mAb) IC16 according to the protocol of the manufacturer (direct IP method) and added to the samples. IC16 recognizes residues 1–16 of the human Aβ sequence and it was used to a final 1:100 concentration [31]. Briefly, total Aβ was immunoprecipitated from soluble brain lysates by mixing fivefold concentrated detergent buffer (50 mM HEPES [pH 7.4], 150 mM NaCl, 0.5% [*v*/*v*] Nonidet P-40, 0.05% [*w*/*v*] SDS and protease inhibitor cocktail [Roche Applied Science]) with each sample. After overnight incubation at 4 °C, samples were washed three times in 1× PBS, 0.1% (w/v) BSA, and once in 10 mM Tris–HCl, pH 7.5. After heating the samples to 95 °C in 15 μL sample buffer (0.36 M Bis–Tris, 0.16 M bicine, 1% [w/v] SDS, 15% [w/v] sucrose, and 0.0075% [w/v] bromophenol blue) the supernatants were loaded in “Urea-SDS-PAGE” section.

### Urea-SDS-PAGE

Immunoprecipitated Aβ peptides from soluble fractions were separated in a 0.75 mm 10% polyacrylamide 8 M urea SDS-gels as described [26, 32]. For separation of Aβ*x*–40 from Aβ*x*–42, a final concentration of 0.3 M H_2_SO_4_ was used in resolving gels. Peptides were transferred to an Immobilon‑P PVDF membrane via semi-dry western blotting (Bio-Rad) at 47 mA for 30 min. Membranes were boiled for 3 min in PBS and then blocked in 5% powder milk in TBST (20 mM Tris, 137 mM NaCl, 0.1% [*v*/*v*] Tween-20) for 30 min afterwards. IC16 antibody was used for overnight immunostaining. After washings in TBS-T, membranes were incubated with appropriate secondary anti-mouse antibody (1:5000, Sigma). Immunoreactive bands were visualized using an ECL enhanced chemiluminescence system (Millipore, MA, USA).

### SDS-PAGE

Pooled samples, soluble and insoluble fractions were prepared in SDS loading buffer (4X Roti-Load, Carl Roth, Germany) and boiled at 95 °C. Protein extracts were separated by SDS-PAGE, transferred onto nitrocellulose membranes (Amersham Hybond ECL), and then blocked in 5% (*w*/*v*) milk powder in TBST. Soluble fractions were used to detect total soluble N-APP (sAPP), using an anti-N-terminal APP antibody (22C11, MAB348, Millipore). Moreover, the following antibodies were used for detection in insoluble fractions: anti-CT15 1:10,000 (APP and CTFs) [33], anti-PSEN1 (7H8, C-terminal), anti-Nicastrin (N1660, Sigma), anti-ADAM10 (Ab19026, Millipore, Massachusetts, USA), anti-Notch1 (ab27526, Abcam), anti-Notch intracellular domain (NICD, New England Bioscence #2421S), and anti-mep1b (AF2895, R&D Systems, Minneapolis, USA).

### Sandwich enzyme-linked immunosorbent assay (ELISA)

Human Aβ1–40 and Aβ1–42 concentrations were measured using commercially available ELISA kits (IBL International, Hamburg, Germany) according to the manufacturer’s protocol. Briefly, soluble or insoluble fractions were diluted 1:50 or 1:200, respectively, then added to wells coated either with anti-human Aβ*x*–42 (44A3) or Aβ*x*–40 (1A10) and incubated overnight at 4 °C. Wells were washed and incubated with HRP-conjugated anti-human Aβ1–*x* (82E1) for 1 h. After final washing steps, TMB solution was added as a substrate. The reaction was stopped with 1N H_2_SO_4_. Optical density (OD) values were measured at 450 nm on a microplate reader (Anthos 2010). For total Aβ*x*–40/42 levels including N-terminally truncated peptides, samples were diluted 1:10 and the ELISA was performed according to the manufacturer’s instructions of LEGEND MAX™ β‐Amyloid x‐40 ELISA Kit (Biolegend, Cat. no.: 842301) and LEGEND MAX™ β‐Amyloid x‐42 ELISA Kit (Biolegend, Cat. no.: 842401).

### Immunohistochemistry on FFPE mouse brain sections

15-month-old mice were sacrificed by cervical dislocation; right hemispheres were washed in 0.01M PBS and immerged in 4% PFA 0.01M PBS for fixation for 48 h. Tissue was dehydrated and embedded in paraffin. Immunohistochemistry was performed on 4 µm sagittal paraffin sections as previously described [34]. In brief, sections were deparaffinized in xylene and rehydrated in a descending series of ethanol. To block endogenous peroxidases, we treated the sections with 0.3% H_2_O_2_ in PBS and antigen retrieval was achieved by boiling sections in 0.01 M citrate buffer pH 6.0, followed by 3-min incubation in 88% formic acid. Non-specific binding sites were blocked by treatment with 4% skim milk and 10% fetal calf serum in PBS for 1 h prior to the addition of the primary antibodies. We used the following antibodies: rabbit anti-polyclonal antibody 77 (pAb77, 1:500) [35], raised against residues 2–14 of the Aβ peptide, detecting N-truncated Aβ peptides (Aβ2–*x*); mouse anti-mAb80C2 (1:1000, 218 231 Synaptic Systems) detecting Aβ1–*x* peptides [36], and mouse anti-GFAP (1:500, 173 004 Synaptic Systems). Primary antibodies were incubated overnight in a humid chamber at room temperature followed by incubation with corresponding biotinylated secondary antibodies (1:200, Dianova). Staining was visualized using the ABC method using a Vectastain kit (Vector Laboratories, Burlingame, USA) and 3,3ʹ-diaminobenzidine (DAB) as chromogen providing a reddish-brown color. Counterstaining was carried out with hematoxylin. In case of Aβ load analysis, the counterstaining was omitted. Serial images were captured with an Olympus B*x*–51 microscope equipped with a Moticam pro 282 camera (Motic, Wetzlar, Germany) on three sections per animal, which were at least 50 µm afar from each other.

### Morris water maze

For behavior analysis, 7-month-old female mice of were tested (*n* = 5–10 animals/group). Spatial learning and memory were assessed by the Morris water maze hidden platform task performed as previously described [37] with minor modifications. Briefly, water maze (120 cm) was filled with clear water (23 ºC). Prominent symbols around the maze provided abundant extra-maze cues. The platform stayed in the same quadrant from day 1 to 4 and the animals were released from four different positions at the pool perimeter. Mice performed four trials per day on 4 consecutive days with a maximum length of 90 s and an inter-trial interval of 90 s. If mice did not find the platform within the given time, they were guided to the platform. Mice were allowed to stay on the platform for 10 s. On the 5th water maze day, a probe trial (60 s) without platform was performed. Basal motor activity was evaluated by swim speed. Learning was assessed by measuring the escape latency to find the platform. Memory capabilities were characterized by the number each mouse crossed the former platform location at probe trial and the latency to reach the location. For vision abilities, a visible platform task was done after the learning assessment at day 6. It consisted of three trials in a row starting the mice opposite to the platform which was indicated by a table tennis ball 15 cm above the platform.

### Monitoring of behavior

A computerized video recording system registered moving-path and duration in water maze tests automatically. The hardware consisted of an IBM-type AT computer combined with a video digitizer and a CCD video camera. The software used for data acquisition and analysis was EthoVision XT^®^ release 8.0 (Noldus Information Technology, Utrecht, the Netherlands).

### Quantification and statistical analysis

All graphs and statistical analyses were prepared using GraphPad Prism 6 software (La Jolla, CA). Western blots were quantified by densitometry using ImageJ v.1.52 (NIH, USA) and data were analyzed by *t* test. For human and mouse brain immunohistochemical analysis, all statistical comparisons were made using two-tailed unpaired Student’s *t* test. Using ImageJ, Aβ load and GFAP staining pictures were binarized to 8-bit images and a fixed intensity threshold was applied defining the DAB staining. Measurements were performed for a percentage area covered by DAB staining. Behavioral data were analyzed by two-way analysis of variance (ANOVA) with genotype factor one and time factor two by repeated measures. Other parameters without repeated measures were analyzed by one-way ANOVA followed by Tukey’s post hoc test. For the analysis, *p* values less than or equal to 0.05 were considered as statistically significant.

## Results

### Meprin β protein levels are increased in sporadic AD brain

**Fig. 1:**
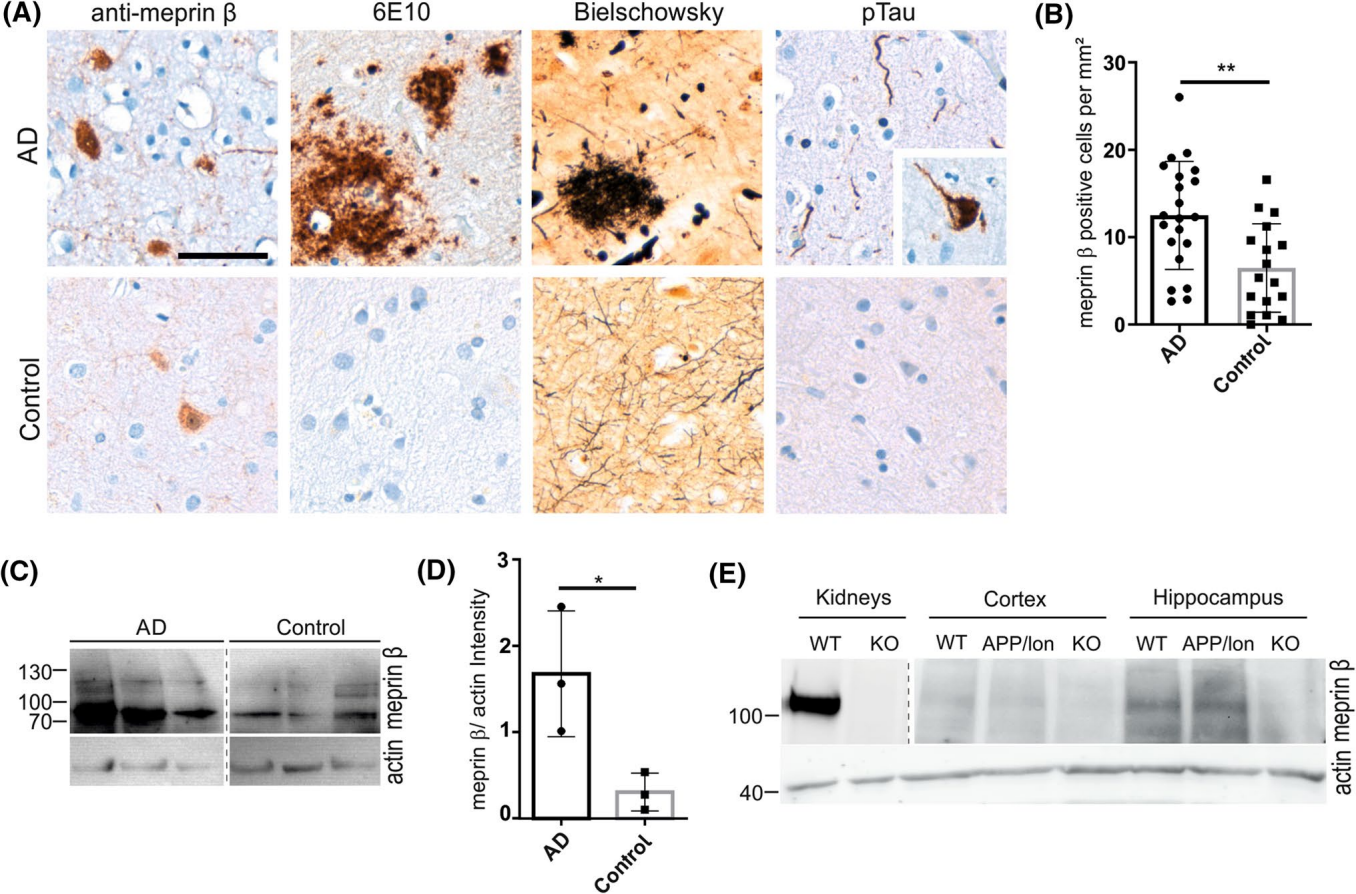
Meprin β expression in brains of AD patients. **A** Representative images of human brain sections from an AD patient and an age-matched, non-demented individual (control). Immunostainings were performed with anti-meprin β antibody, anti-Aβ1–16 (6E10), for the detection of Aβ aggregates, Bielschowsky silver stain, which impregnates both amyloid senile plaques and neurofibrillary tangles, and anti-phosphorylated Tau protein (AT8) antibody. Scale bars = 100 µm. Note Aβ accumulation, neuritic plaques and pTau stainings confirming AD diagnosis. **B** Densitometric analysis of meprin β-positive neurons in cortical sections revealed a significantly higher number of meprin β positive neurons in AD compared to control brains from non-AD individuals. Graph shows mean ± SEM (*n* = 21 AD, *n* = 17 Control; **p <  0.05, *t*-test). Representative comparison of all cases analyzed can be found in supplementary Fig. S2. **C** Human brain lysates were tested with a polyclonal anti-meprin β antibody (supplementary Fig. S1) and actin as loading control. Control and AD samples were on the same gel but are rearranged for better presentation. **D** Quantification of meprin β protein levels normalized to actin levels of AD versus control brains. Graph shows mean ± SEM (*n* = 3 AD, *n* = 3 Control; *p* < 0.05 *t*-test). **E** Mouse kidney and different brain region samples were tested using anti-meprin β antibodies in Western blot analysis. Signals of meprin β were detected in cortex and hippocampus of wild-type and APP/lon mice. Analysis of meprin β knockout kidney and brain samples confirmed the absence of meprin β in the APP/lon × *Mep1b*^*−/−*^ mice (KO). Kidney and brain samples were on the same gel but they had highly different intensities and were arranged for better presentation (dotted line)

We previously identified the metalloprotease meprin β as an APP-cleaving enzyme with β-secretase activity. Therefore, we wanted to investigate, whether meprin β brain protein levels differ between healthy control and AD patients. Herein, we performed immunohistochemical staining of human brain sections using anti-6E10, Bielschowsky and anti-tau to confirm the diagnosis of AD patients showing amyloid plaques and neurofibrillary tangles (Fig. 1A). Upon immunohistochemical assessment, a subpopulation of neurons in cortical layers III to VI showed prominent DAB signal of soma and neurites in all samples and was thus defined as meprin β positive. Generation and specificity of meprin β antibodies are described in the supplementary information (Fig. S1). Immunohistological analyses of sections of frontal cortex show an increase in the number of meprin β-positive cortical neurons in AD (*n* = 21) compared to control brains of non-demented patients (*n* = 17) (Fig. 1A, B) of nearly 50% (see Supplementary Fig. S2, for all cases). Additionally, we performed western blot analysis with brain tissue homogenates of sporadic AD patients (*n* = 3) versus age matched non-demented controls (*n* = 3) (Fig. 1C). We found significantly increased signals of meprin β in brains of AD patients compared to controls (Fig. 1C, D). Moreover, we were able to detect meprin β in the brains of wild-type and in the AD-mouse model APP/lon mice using western blot analysis. Interestingly, expression of meprin β seems to be higher in the hippocampus compared to cortex (Fig. 1E). In summary, the morphometric analysis revealed a significant up-regulation of meprin β specifically in AD compared to controls, thus confirming previous observations by Schlenzig and colleagues [27].

### Aβ levels are reduced in APP/lon mice with a knockout for meprin β

**Fig. 2:**
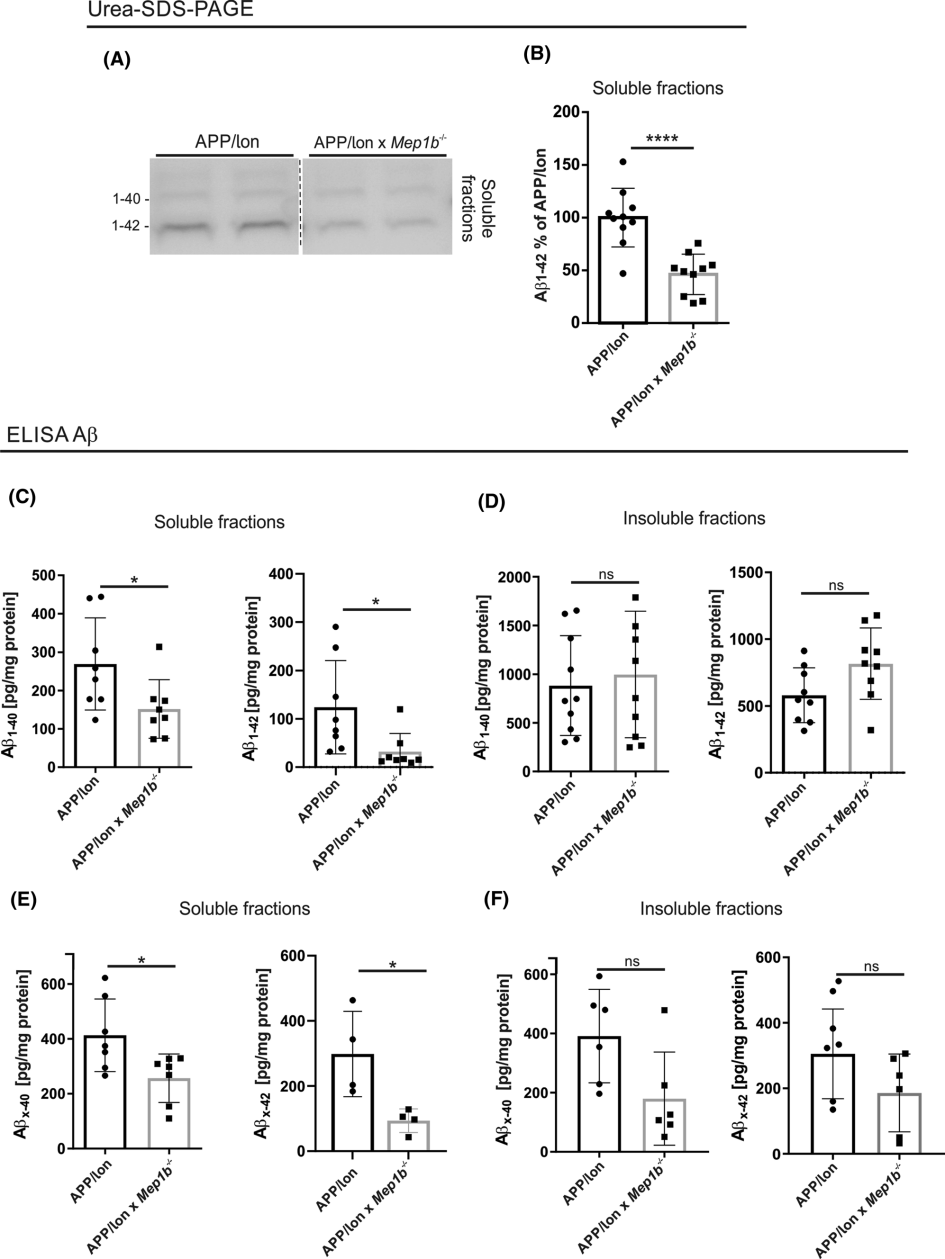
Aβ levels are reduced in APP/lon mice knockout for meprin β. **A** Soluble-extracted brain fractions from 15-month-old APP/lon and APP/lon × *Mep1b*^−/−^ mice were analyzed by immunoblot for Aβ peptides species. Samples were on the same gel but are rearranged for better presentation. **B** Densitometric analysis of Aβ_1–42_ levels show a decrease in soluble fractions (****p < 0.0001, *t*-test, *n* = 10). **C** ELISA analysis showed decreased levels of 1–40 (*p < 0.05, *t*-test, *n* = 8) and 1–42 (*p < 0.05, *t*-test, *n* = 8) in soluble fractions of APP/lon × *Mep1b*^−/−^ compared to APP/lon mice. **D** No differences were detected in insoluble brain fractions (*n* = 9–10). **E** A following ELISA approach revealed reduced levels of Aβ *x*–40 (*p* < 0.005, *t*-test, *n* = 7) and *x*–42 (*p* < 0.05, *t*-test, *n* = 4) in soluble brain fractions of APP/lon × *Mep1b*^−/−^ **(F)** No obvious differences were identified in total Aβ species *x*–40 and *x*–42 in insoluble brain fractions (*n* = 6)

Given that meprin β can generate N-terminally truncated Aβ peptides in vitro [24], we hypothesized that Aβ levels would also be altered by meprin β knockout in vivo. To test this assumption, *Mep1b*^−/−^ mice were crossed to an APP-overexpressing mouse model. We have shown previously that meprin β generates predominantly N-terminal truncated Aβ2–*x* peptides, which are fully blocked when APP carries the Swedish familial mutation close to the β-secretase cleavage site [26]. Since most commonly used AD mouse models harbor the Swedish mutation in the APP sequence, these mice are not a useful model to investigate the generation of N-terminally truncated Aβ2–*x* peptides by meprin β. Hence, Mep1b^−/−^ animals were crossed into mice expressing APP only with a ‘London’ mutation (V717I) close to the γ-secretase cleavage site. This mutation also increases the amount of Aβ*x*–42 but leaves the N-terminal region of the Aβ sequence unaltered. This model reflects more appropriately the situation for sporadic AD in terms of the accessibility and involvement of enzymes close to the N-terminus of Aβ. Brains from 15-month-old APP/lon and APP/lon × *Mep1b*^−/−^ mice were dissected and processed to isolate soluble (PBS) Aβ fractions. To analyze the meprin β-dependent generation of Aβ peptides we applied two different detection methods. First, we performed an immunoprecipitation assay followed by Urea-SDS-PAGE (Fig. 2A, B). For the purpose of detecting different Aβ species we used here the IC16 antibody. A significant decrease in levels of Aβ was detected in soluble fractions of APP/lon mice lacking *Mep1b* (*n* = 10), compared to APP/lon mice (*n* = 10) (Fig. 2A, B)*.*

In a second approach, we used sandwich enzyme-linked immunosorbent assays (sELISA) for the specific detection of Aβ1–40 and Aβ1–42 (Fig. 2C, D), followed by another ELISA to detect total Aβ*x*–40 and Aβ*x*–42 levels (Fig. 2E). We detected a remarkable reduction in levels of soluble Aβ1–40 and Aβ1–42 in the brains of APP/lon × *Mep1b*^−/−^ compared to APP/lon mice alone (*n* = 8 animals/group). However, the Aβ1–40 and Aβ1–42 concentrations observed in the insoluble fractions were not significantly altered amongst the different genotypes (Fig. 2D). We have previously shown that meprin β generated Aβ2–*x* peptides exhibit a higher aggregation rate than Aβ1–*x* [26]. Hence, we would expect a reduction of Aβ2–*x* peptides in insoluble fractions of APP/lon × *Mep1b*^−/−^ mice. However, the antibody 82E1 used in this setup specifically detects the very N-terminus of Aβ peptides (1–*x*) generated by BACE1 cleavage [38]. Recognition of Aβ1–40 and Aβ1–42, but not Aβ2-40, was further confirmed by immunoblot of 82E1 against synthetic peptides (Fig. S3). Reprobing of the same membrane with the IC16 antibody revealed the Aβ2-40 detection.

The amounts of Aβ*x*–40 and Aβ*x*–42 were even more strongly decreased when meprin β was lacking compared to APP/lon mice alone (Fig. 2E). In general, we observed that the absolute levels of *x*–40 and *x*–42 detected were almost twofold higher than the corresponding Aβ1–40 and Aβ1–42 (Fig. 2C and E for comparison). Therefore, we conclude that the remaining portion of *x*–40 and *x*–42 might correspond to all species of N-terminally truncated Aβ peptides, which have been documented previously [16, 24] No significant differences could be detected in insoluble fractions for Aβ*x*–40 or Aβ*x*–42 (2F)

### General APP processing and secretase activities are not altered in APP/lon mice lacking meprin β

**Fig. 3:**
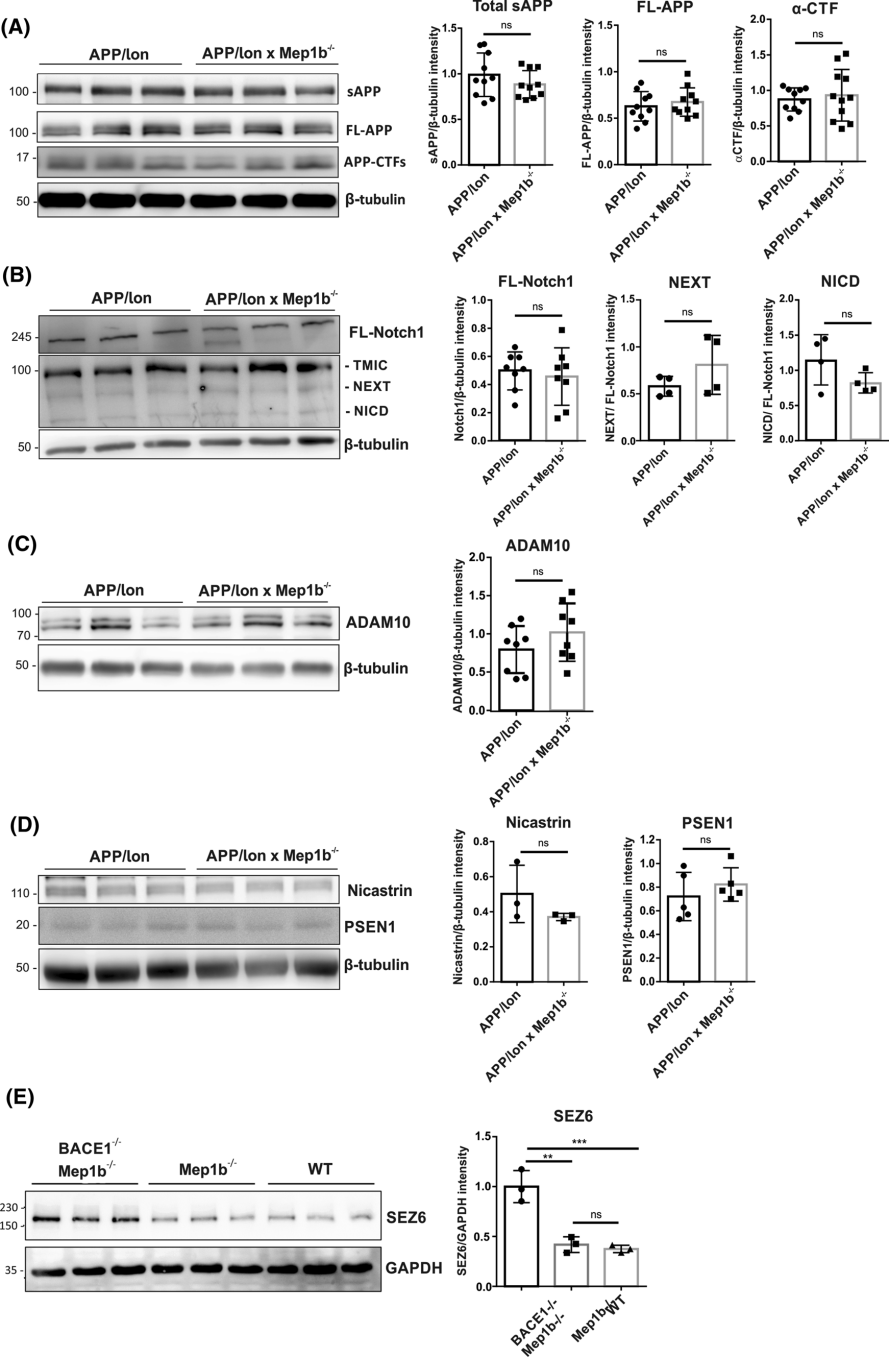
The meprin β knockout has no evident effect on the overall APP processing and its secretases. **A** Soluble brain fractions of 15-month-old APP/lon and APP/lon × *Mep1b*^−/−^ mice (*n* = 10/group) were analyzed by immunoblot for sAPP forms using the monoclonal antibody 22C11. Further immunoblot analysis of insoluble brain fractions using an antibody against the C-terminus of full-length APP and CTFs in 15-month-old APP/lon and APP/lon × *Mep1b*^−/−^ mice (*n* = 10/group). **B** Immunoblot analysis shows protein levels for Notch1 and (*n* = 8/group) its fragments (*n* = 4/group). **C** The protein levels for ADAM10 in insoluble brain fractions show no differences between the groups (*n* = 8/group). **D** γ-Secretase subunits PSEN1 and Nicastrin in insoluble fractions of APP/lon and APP/lon × *Mep1b*^−/−^ mice (*n* = 5/group). **E** Total brain lysates from double knockout mice for BACE1 and meprin β, as well as *Mep1b*^−/−^ and WT mice were analyzed by immunoblot for Sez6. **A–D** Densitometric analyses normalized to β-tubulin revealed no significant differences. **E** Densitometric analysis normalized to GAPDH detected an increase in levels of endogenous Sez6 in the double knockout mice lacking BACE1, but no significant differences were found in meprin β knockout compared to WT brain homogenates

Previously, we have shown that meprin β participates in the processing of APP in vitro, generating soluble N-APP fragments and Aβ peptides [24, 39]. To evaluate the impact of meprin β on general APP processing in vivo, we analyzed both soluble and insoluble brain fractions of 15-month-old APP/lon and APP/lon × *Mep1b*^−/−^ mice. In soluble fractions, analysis by SDS-PAGE and subsequent western blotting revealed no obvious differences in total sAPP (Fig. 3A). Similar, in insoluble fractions, full length unprocessed APP or CTFs levels were unaltered between the two groups (Fig 3A). This result is in part consistent with BACE1 knockout data, where also no measurable effect on full-length APP expression was detected [40, 41].

As γ-secretase is considered a key target for Aβ generation and previous work showed meprin β influence on α-secretase [42], we wanted to investigate whether the knockout of meprin β may lead to decreased secretases activities. Notch1 is a type I transmembrane protein involved in numerous pathways such as cell fate and morphogenesis in both embryonic and adult brain [43]. Upon ligand binding, Notch1 is processed initially by ADAM10, by cleavage at its extracellular juxtamembrane domain. This is followed by intramembrane cleavage through γ-secretase, which generates the cytoplasmic domain of Notch (NICD). We analysed the production of these two fragments in the insoluble fraction by western blot. Loss of meprin β did not change the processing or levels of Notch1 (Fig. 3B), indicating that the metalloproteinase has no significant effect on α- or γ-secretase activity in this model. ADAM10 protein levels were evaluated in insoluble fractions and results confirmed there were no significant difference between the two groups (Fig. 3C).

γ-Secretase is a multiprotein complex consisting of Presenilin (PSEN), Nicastrin, Aph-1, and Pen-2 and all four proteins are necessary for full intramembrane proteolytic activity [44]. Exemplary we evaluated γ-secretase expression through PSEN1 and Nicastrin levels, which showed no significant difference between APP/lon (*n* = 5) and APP/lon × *Mep1b*^−/−^ (*n* = 5) mice (Fig 3D). In sum, the meprin β knockout has no significant effect on the expression of APP cleaving secretases.

Having ruled out alterations in α- and γ-secretase activities with regard to APP processing, we wanted to investigate whether the knockout of meprin β may lead to decreased BACE1 activity. As the majority of Aβ generation has been attributed to BACE1, lower Aβ levels observed in APP/lon × *Mep1b*^−/−^ mice could be due to decreased BACE1 activity. To investigate this, we analyzed the well-described BACE1 substrate Sez6, which is involved in maintenance of dendritic arborisation and enhancing synaptic connectivity [45]. The full-length Sez6 is cleaved by BACE1 and it generates a secreted soluble ectodomain and a C-terminal transmembrane fragment, which is further cleaved by γ-secretase [11]. To evaluate whether meprin β affects BACE1 activity, we analyzed the meprin β knockout effect on processing of Sez6. Therefore, we used total brain lysates from *Mep1b*^−/−^ mice, double knockout mice for BACE1 and meprin β compared to wild-type (WT) mice. As expected, increased levels of Sez6 were detected in the double knockout mice lacking BACE1. In contrast, levels of endogenous Sez6 from meprin β knockout brains remained the same as in WT animals (Fig. 3E), suggesting that BACE1 activity is not altered in the absence of meprin β.

### N-terminally truncated Aβ2–*x* peptide deposition is decreased in APP/lon mice lacking meprin β

**Fig. 4:**
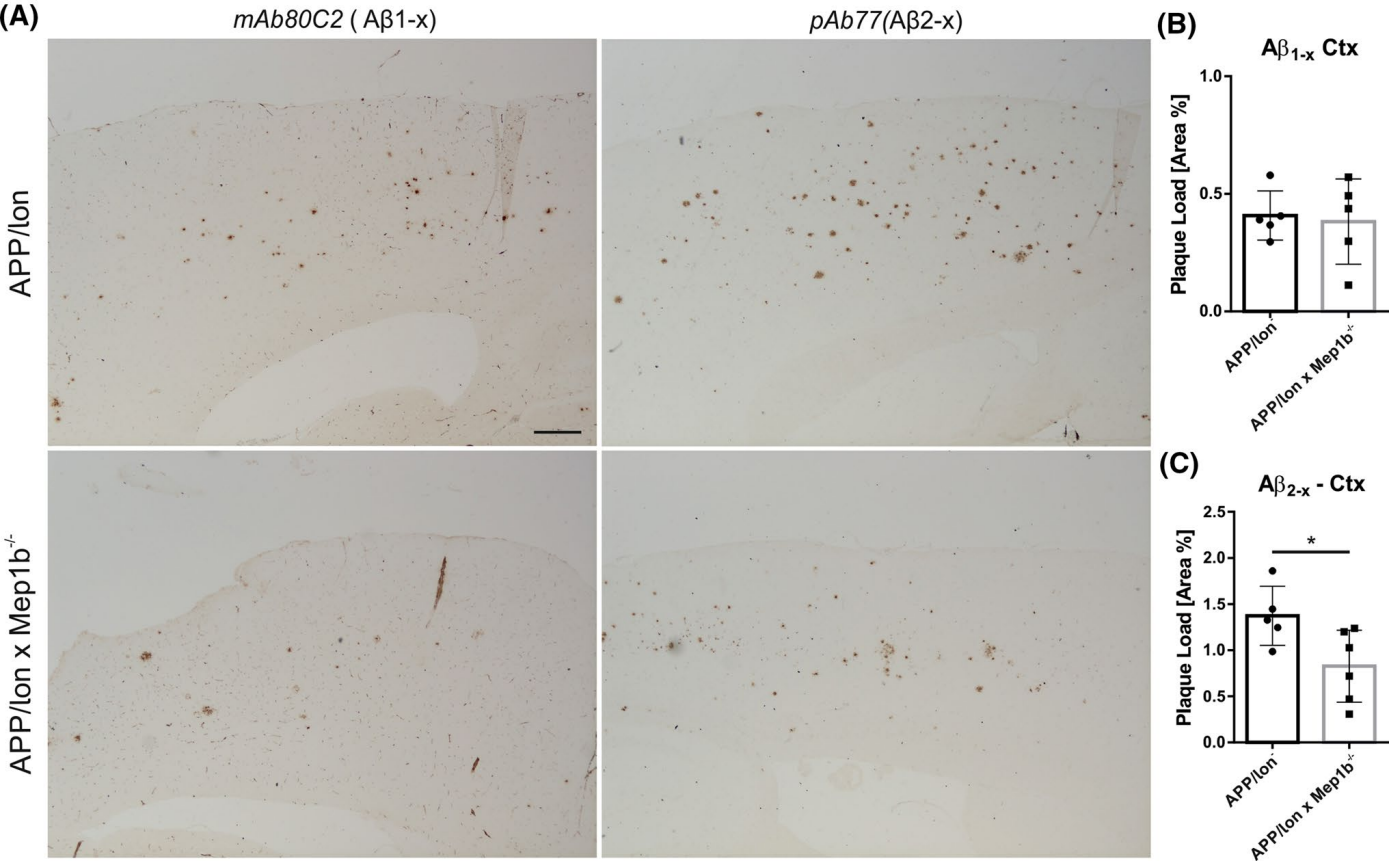
N-terminally truncated Aβ2–*x* peptide deposition is decreased in APP/lon mice lacking meprin β. **A** Representative image of cortices of sagittal brain sections from 15-month-old APP/lon and APP/lon × *Mep1b*^−/−^ which were embedded in paraffin and stained for Aβ1–*x* (mAb 80C2) and Aβ2–*x* (pAb77) plaque load. Detection was made possible through hydrogen peroxidase and 3,3ʹ-diaminobenzidine (DAB) reaction. **B** Quantitative analysis showing the percentage of stained area for Aβ1–*x* peptides (*n* = 5). **C** Quantitative analysis showing percentage of stained area for N-terminally truncated Aβ2–*x* peptides (*p < 0.05, *t*-test, *n* = 5–6). Scale bar = 100 µm

Meprin β is capable of generating Aβ2–*x* peptides in vitro [24, 26]. Subsequently we wanted to determine whether the *Mep1b* knockout had any effect on Aβ2–*x* deposition in vivo. Thus, we evaluated plaque pathology in brains of APP/lon mice using antibodies specifically detecting Aβ peptides starting at position p1 or p2. Amyloid plaques arise in APP/lon mice at the age of 10–12 months [28]. To analyze plaque deposition, sagittal brain sections of 15-month-old APP/lon and APP/lon × *Mep1b* mice were stained using pAb77, specifically detecting Aβ2–*x* and mAb 80C2, which binds to Aβ1–*x*. The selectivity of the Aβ2–*x* (Aβ2–*x* pAb77) and Aβ1–*x* (80C2) antibodies have been confirmed pr^−/−^eviously [35, 36]. In general, both antibodies detected mainly diffuse plaques of different sizes in cortex and hippocampus. While we observed amyloid plaques spread throughout all cortical layers, plaques in the hippocampal formation seemed to be restricted to CA1 and subiculum. Similarly to AD patients, the subiculum is the first brain region to present amyloid deposits in APP/lon mice [46]. A significant decrease in Aβ2–*x* plaque load of about 40% was detected in the cortex of APP/lon mice lacking *Mep1b* compared to APP/lon mice alone (Fig. 4A, 4C). However, no significant difference between the two groups was evident for Aβ1–*x* staining (Fig. 4A, B). This result correlates well with the observed effect on insoluble Aβ1–40 and Aβ1–42 levels documented by ELISA (Fig. 2). Taken together, these results indicate that meprin β is involved in the generation of soluble Aβ peptides in vivo and that it has an impact on plaque deposition through the generation of Aβ2–*x* peptides.

### Reactive astrocytes are decreased in the cortex of APP/lon × *Mep1b*^−/−^ mice

**Fig. 5:**
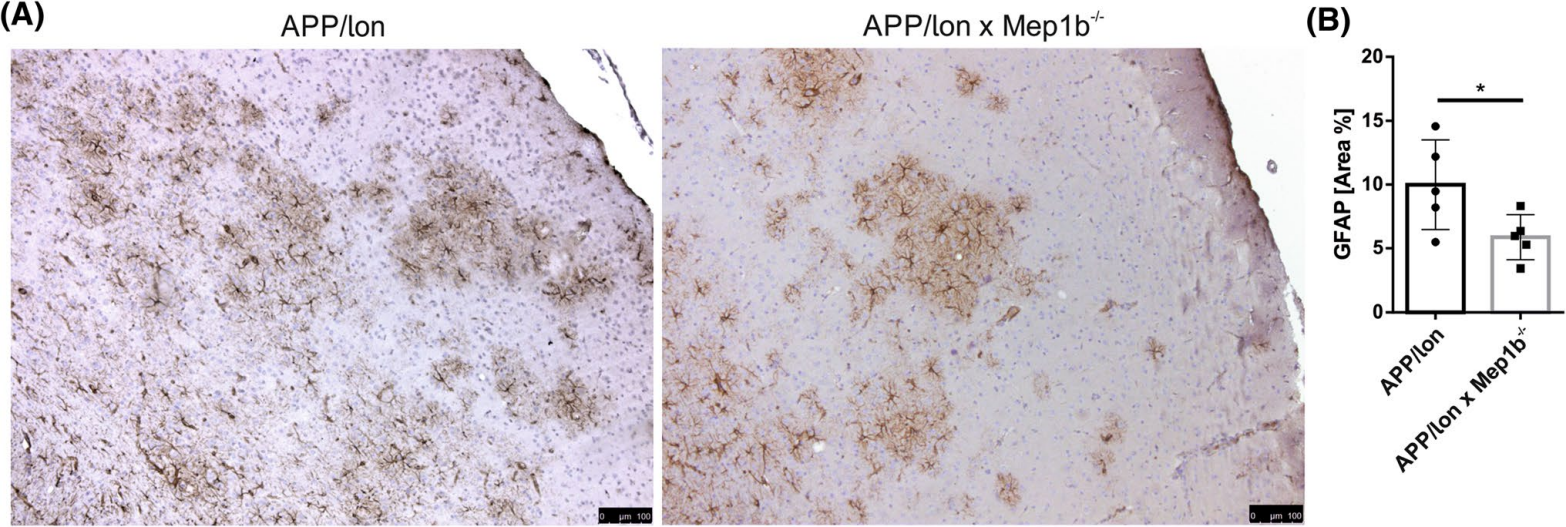
GFAP immunoreactivity is increased in the cortex of APPlon × *Mep1b*^−/−^ mice. **A** Representative image of sagittal brain sections from 15-month-old APP/lon and APP/lon × *Mep1b*^−/−^ embedded in paraffin and stained for GFAP and hematoxylin. **B** Quantitative analysis showing the percentage area covered by DAB staining (*p < 0.05, *t*-test, *n* = 5/group). Scale bar = 100 µm

Severity of AD pathology strongly correlates with the density of activated astrocytes [47]. Numerous AD mouse models have demonstrated a correlation of astrocytic gliosis with plaque pathology [48, 49]. Such gliosis can be identified by staining of glial fibrillary acidic protein (GFAP) in reactive astrocytes surrounding amyloid plaques [50]. Since we have demonstrated decreased levels of soluble Aβ peptides and reduced Aβ2–*x*-derived extracellular plaques, we sought to determine whether gliosis in the brains of APP/lon × *Mep1b*^−/−^ was also reduced. We analyzed cortical sections immunohistochemically stained for GFAP in 15-month-old APP/lon and APP/lon × *Mep1b*^−/−^ mice. Images revealed extensive gliosis throughout the cortex of APP/lon mice (Fig. 5A) thus confirming previous work [48]. However, APP/lon × *Mep1b*^−/−^ mice showed a significant decrease in the area of GFAP-positive immunostaining (Fig. 5B). This result further strengthens our observation that decreased meprin β expression reduces Aβ-peptide generation and subsequently improves brain pathology.

### Meprin β knockout rescues learning behavior impairments in APP/lon mice

**Fig. 6:**
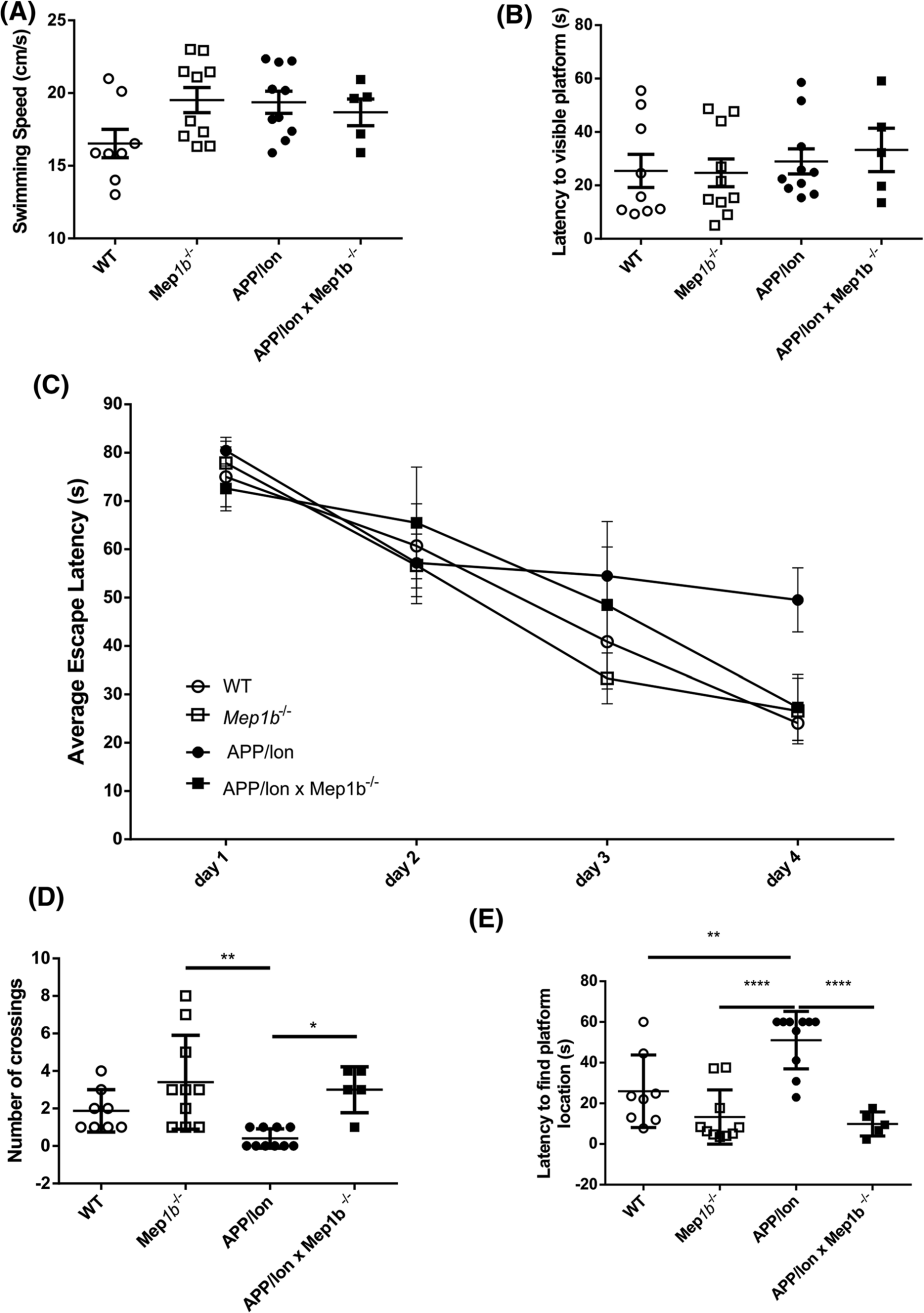
Meprin β knockout rescues cognitive impairments in APP/lon mice. Spatial learning and memory tests were carried out on 7-month-old APP/lon, APP/lon × *Mep1b*^−/−^, *Mep1b*^−/−^, and wild-type mice using the Morris water maze setting. **A** Swimming speed analysis showed no motor impairment in any of the groups. **B** Latency to find visible platform was evaluated after probe trial and no specific deficits were detected. **C** The average escape latency in each trial was measured for 4 days and 24 h later all experimental groups were subjected to a probe trial (PT) in which the platform was removed. On day 4, APP/lon showed significant learning deficit compared to WT mice (*p < 0.05) and to *Mep1b*^−/−^ (*p < 0.05), but not to APP/lon × *Mep1b*^−/−^ mice. Data shown is the mean ± SEM of four different trials performed on day 4. Statistical analysis performed with one-way ANOVA followed by Tukey’s post hoc test. **D** Memory was evaluated by measuring the numbers of crossings over the former platform location. APP/lon mice showed significant differences when compared to *Mep1b*^−/−^ or APP/lon × *Mep1b*^−/−^ (*p < 0.05, **p = 0.0016). **E** Latency to reach platform location in all four groups. On PT day, cognitive deficits are prominent in APP/lon when compared to WT (*p < 0.05), Mep1b-/- (****p < 0.0001) and APP/lon × *Mep1b*^−/−^ mice (***p < 0.0001). Data are presented as mean ± SEM (*n* = 5–10). Graph shows latency to find platform location in one probe trial

As we have demonstrated that absence of meprin β has no detectable influence on other APP processing enzymes, but significantly alters amounts of soluble Aβ peptides, which are strongly associated with a decline in cognitive ability, we tested whether meprin β knockout had any influence on learning behavior of APP/lon mice. As control groups, we included age-matched WT and *Mep1b*^−/−^ mice. In APP/lon mice, behavior alterations start at the age of 6 months [28]. To assess hippocampus-dependent learning we chose the Morris water maze hidden platform task [37]. All genotypes showed the same basal motor activity indicated by unaltered swimming speed (*F*(3;29) = 2.489, *p *= 0.0801) (Fig. 6A) and had no specific deficits of finding a visible platform (*F*(3;29) = 0.3073 *p *= 0.8199) in the pool (Fig. 6B), allowing for evaluation of cognitive performance.

As expected, APP/lon mice showed a significant learning deficit compared to wild-type (WT) mice (Fig. 6C) (*p *< 0.05 on day 4). Escape latency to find the visible platform from day 4 shows also a significant difference when comparing APP/lon to *Mep1b*^−/−^ mice (day 4 *p *< 0.05) WT mice showed no different escape latency when compared to either APP/lon × *Mep1b*^−/−^ or *Mep1b*^−/−^. Memory abilities were characterized on the probe trial (PT) by the number each mouse crossed the former platform location and the latency to reach the location. In line with the observations on learning, APP/lon mice showed the fewest crossings of the former platform location (Fig. 6D) [*F*(3;29) = 6.587, *p *= 0.0016], showing significant differences to all other genotypes (*p *< 0.05). Most interestingly, on PT day, APP/lon mice showed the highest average latency [*F*(3;29) = 15.32, *p *< 0.0001] to find the platform location and with significant difference to all other three genotypes (Fig. 6E). This effect is also seen when comparing APP/lon to APP/lon × *Mep1b*^−/−^(*p *< 0.0001). Taken together, these findings indicate that the absence of meprin β in APP/lon mice ameliorates the cognitive impairments since there were no differences observed in learning behavior when compared to WT animals. APP/lon mice deficient for meprin β were able to learn the location of the hidden platform, and to find the platform location when it was removed. Hence, the present data suggest that meprin β contributes to the development of cognitive impairments in APP/lon mice.

## Discussion

Generation of Aβ peptides is a hallmark of AD pathology. BACE1 was identified as the major β-secretase, however, so far, all therapeutic attempts to block BACE1 activity to hold AD progression or cure the symptoms in AD patients have not been successful. There are many possible explanations, one of which is the existence of alternative proteases acting complementary to BACE1. Here we demonstrate that loss of the alternative β-secretase meprin β is capable of rescuing the learning deficits in the APP/lon AD mouse model. Importantly, most AD mouse models carry the APPswe mutation, which is a variant that is highly prone to BACE1 activity but strongly impairs meprin β cleavage [26]. We hypothesize that the wide use of APPswe models in the Alzheimer field opened just a very small window to investigate alternative amyloidogenic APP processing. Of note, the potential relevance for meprin β in AD was recently supported by a genetic study where a *Mep1b* variant was identified as one of the AD-associated genes in a British dementia cohort [23].

Previously, we and others have identified APP as a substrate for meprin β in vitro [27, 39]. Moreover, mass spectrometry analysis of peptides incubated with meprin β revealed cleavage sites not only at the aspartate p1, but also in alanine p2 and the peptide bond in p3, resulting in an N-terminal glutamate residue [24]. Together, these observations identify meprin β as a protease candidate for the generation of not only the canonical full-length Aβ peptides, but also N-terminally truncated Aβ2–*x* and Aβ3–*x* species commonly found in AD brain. Of particular interest, Aβ3–*x* generated by meprin β serve as a substrate for glutaminyl cyclase (QC) which converts the N-terminus into pyroglutamic acid to produce pyroglutamate Aβ peptides (pGlu-3Aβ) [27]. This particular peptide is found in amyloid plaques in abundance, comprising 15–45% of total Aβ in AD brains [51]. Preclinical immunotherapy aiming at the reduction of pGlu-3Aβ have shown remarkable results in cognitive improvement of AD-like mouse models [52, 53]. Recently Eli Lilly & Company announced that the Phase 2 TRAILBLAZER-ALZ study using “Donanemab”, a monoclonal antibody that recognizes pyroglutamated Aβ, slowed cognitive decline in people with early AD. To provide further evidence for the relevance of meprin β in vivo, we generated mouse lines to analyze whether meprin β plays an important role in the pathogenesis of AD. The present work describes the in vivo effect of meprin β gene knockout on APP processing, Aβ generation and behavioral phenotype in APP/lon mice.

The APP/lon animal model is characterized by an age-dependent, progressive increase of soluble Aβ levels [28]. Interestingly, homozygous knockout of meprin β led to a decrease in soluble Aβ in this mouse model. In APP/lon mice, the absolute levels of Aβ*x*–40 and *x*–42 detected by ELISA were around twofold higher than the corresponding specific levels of Aβ1–40 and Aβ1–42. Hence, the remaining amount of *x*–40 and *x*–42 likely corresponds to all species of N-terminally truncated Aβ peptides. [38]. N-terminal truncations make up the majority of Aβ species in AD [12, 54] but, of note, are much less abundant in the transgenic mouse models that overexpress APPswe, which might explain the differences in amyloid deposition mechanisms between human and rodents [55]. There are several known mutations within the APP sequence, but the majority of AD research is based on mouse models harboring the APPswe (K670N/M671L) mutation. However, cells overexpressing meprin β and APPswe showed a lack of Aβ2–*x* variants [26]. This result indicates that meprin β is responsible for generating N-terminally truncated Aβ2–*x* almost exclusively from the APPwt sequences. Essentially, wild-type APP is considered as a relatively poor substrate for BACE1, which cleaves preferentially APPswe [56]. In terms of the relative abundance of specific variants of Aβ peptides, such as Aβ1–42, and N-terminally truncated Aβ2/3–42, clear differences between AD brain and APP23 mice were observed [54, 57], with Aβ1–40 being the most abundant species detected in brain lysates of the latter model.

Previously, we and others detected increased levels of meprin β mRNA in brains of demented versus non-demented control patients [24, 27]. In this study, we could further strengthen this observation by providing evidence for increased meprin β protein expression levels in AD brains. We found significantly elevated amounts of strongly meprin β-positive cells in brains of sporadic AD patients, compared to brains of age-matched non-demented control patients. While cause and relevance of this clearly deserves further studies, increased meprin β-positive cell count (in combination with previously published elevated mRNA levels found in AD) supports our hypothesis of a potential role of meprin β in AD pathology. We already showed a different APP cleavage pattern in brains of meprin β knockout compared to WT mice, suggesting that meprin β is involved in N-terminal processing of endogenous APP in vivo [26]. Moreover, we could clearly show that meprin β is expressed in the mouse brain, particularly in the hippocampus. Although meprin β expression is lower in the brain compared to kidneys, we were able to show that the neural processing of APP is directly affected by its knockout.

It has been shown that there is a difference in the Aβ pattern composition between brain samples from non-demented and demented individuals. Regarding the species composition, soluble Aβ aggregates that accumulate in AD differ from those generated during normal ageing. The higher neurotoxicity (more prone to aggregate) correlates with the predominance of N-terminally truncated species over the full-length forms [58]. Overall, brain tissue of AD patients had more N-terminally truncated and pyroglutamate-modified Aβ accumulation than healthy elderly patients, suggesting that they might play a critical role in plaque formation [59]. In AD temporal lobe samples, Aβ1–42 was the predominant form and the second most abundant species comigrated with synthetic Aβ2-42 [54]. The concentration of soluble Aβ peptides (probably in the form of neurotoxic oligomeric assemblies) is strongly associated with cognitive decline and pathological synaptic changes in neurons [60]. Because the loss of charged amino acids in the Aβ truncated forms contribute to their enhanced insolubility and resistance to enzymatic degradation, they are considered highly neurotoxic and induce faster aggregation. Thus, N-terminally truncated species might be associated with more severe neurodegeneration [58]. For example, N-terminally truncated Aβ4–x peptides seem to be restricted mainly to amyloid plaque cores and cerebral amyloid angiopathy in AD patients and in AD mouse models [61]. Transgenic mice expressing Aβ4-42 (Tg4-42 transgenic line) developed a massive CA1 pyramidal neuron loss in the hippocampus [62]. Aβ4-42 was also identified as the major N-truncated species in postmortem brain samples from aged controls, patients with vascular dementia, and AD patients [63, 64].

AD is characterized by accumulation of different forms of Aβ peptides and their deposition into extracellular amyloid plaques. Plaque deposition in APP/lon mice arise at the age of 10–12 months and it shows patterns that are reminiscent of AD brain. Using an antibody specific to Aβ2–*x* peptides we were able to detect plaque load by immunohistochemistry in aged APP/lon mice in the presence or absence of meprin β. Peptides starting at p2 in the form of small round and diffuse plaques were detected in cortex and hippocampus. More interestingly, a 40% decrease in Aβ2–*x* plaque load was detected in the cortex of APP/lon mice lacking meprin β. However, meprin β knockout decreased but not fully abolished Aβ2–*x* production as we expected. These results further support the idea that in addition to BACE1, meprin β influences the generation of Aβ peptides to some extent. As a diagnostic tool, assessment of Aβ peptides in CSF has been used to support the diagnosis of AD and to identify patients who might be at risk of developing AD. Along with other truncated species, Aβ2–42, for example, is decreased in CSF [65] and enriched in AD brains [16]. Moreover, deficits in learning and memory, as assessed by Morris water maze tasks, were detected at 7 months of age in APP/lon in accordance with a previous report [66]. We now demonstrate that APP/lon mice lacking meprin β showed cognitive abilities similar to wild-type mice. Further accumulation of brain plaques is usually associated with increased brain inflammation that eventually lead to cognitive impairment [67]. Amyloid β increased generation and accumulation lead to an extensive proliferation of reactive astrocytes that can secrete an excess of proinflammatory cytokines [68]. Previous work analyzing astroglial activation demonstrated that GFAP-positive cells were mostly localized surrounding amyloid plaques in aged APP/lon [48]. We have shown here decreased levels of GFAP signal in the absence of meprin β in vivo. This result supports the in vivo effect of meprin β gene knockout on amyloid pathology. Since APP/lon × *Mep1b*^−/−^ mice present decreased levels of soluble Aβ*x*–40 and Aβ*x*–42 and particularly reduced Aβ2–*x* plaque load, the astroglial response is lower compared to APP/lon alone.

Additionally, supporting the relevance in murine AD models, the group of Dennis Selkoe [69] detected meprin β in microsomal fractions from mouse brain lysates that are responsible for the majority of Aβ production. Although a portion of BACE1 co-fractionated with ADAM10 in this higher molecular weight fractions, the majority of BACE1 protein was found in low molecular weight pools that did not contribute to Aβ generation. Instead, meprin β showed a much stronger co-fractionation in the high molecular weight fractions, which may indicate its importance for Aβ generation.

Of note, truncated Aβ fragments favor seeding and aggregation of other Aβ species [70]. Truncated fragments such as Aβ2–*x* peptides generated by meprin β promote and seed aggregation of less prone Aβ species. Although we could not detect obvious differences in Aβ1–*x* plaque deposition, we observed significant reduced Aβ2–*x* plaque load and decreased levels of soluble Aβ1–*x* species. While the presence of extracellular plaque deposition is a hallmark of AD pathology, soluble amyloid β species present much more evidence as a source of primary synaptoxicity and memory loss in transgenic mice [71]. Moreover, plaque deposition is not clearly associated with disease severity. In our conditions, the fact that we could not detect alterations in Aβ1–*x* deposition might be related to the age of animals. While most AD mice models bearing APPswe mutations begin plaque deposition around 2-month-old, APP/lon mice show first signs of amyloid deposition at age of 10 months old. By the age of 15 month-old plaques are more abundant but there are no signs of neurodegeneration yet. It is possible that the full impact of lack of functional meprin β and reduced 2–*x* production will show more pronounced effects at a later age.

We cannot exclude, though, that some N-truncated amyloid β peptides might arise secondarily by exopeptidase activity towards Aβ1–40/42 originally produced by BACE1. For example, it was recently shown that aminopeptidase A has the ability to truncate Aβ1–40 at its N-terminus, thereby yielding Aβ2-40 [72]. Moreover, dipeptidyl peptidase 4 releases the N-terminus of Aβ, converting Aβ1–40 into Aβ3–40 [73]. In both studies, genetic reduction and pharmacological blockade of these enzymes reduced full-length Aβ production. However, many findings point to an important role of meprin β within initial Aβ production. Here we report the generation of the mouse line APP/lon × *Mep1b*^−/−^ which presented improved cognitive abilities and a decrease of total Aβ levels. We believe that our findings offer several new pathways that may improve our understanding of AD pathogenesis and, in particular, roles of previously underestimated molecules and mechanisms therein.

## Supplementary Information

Below is the link to the electronic supplementary material.

Supplementary file 1 (PDF 5256 KB)

## Data Availability

All primary data and materials in the manuscript are available upon reasonable request.
