# Supplementary material for: Meprin β knockout reduces brain Aβ levels and rescues learning and memory impairments in the APP/lon mouse model for Alzheimer’s disease
Source: Cell Mol Life Sci. 2022 Mar 2;79(3):168. doi: 10.1007/s00018-022-04205-5 (PMC8891209; doi:10.1007/s00018-022-04205-5)
Supplement: Supplementary file 1 — (PDF 5256 KB) [file 18_2022_4205_MOESM1_ESM.pdf]

Supplementary Data

Meprin β knockout reduces brain Aβ levels and rescues learning and memory impairments in the APP/lon mouse model for Alzheimer's disease

Liana Marengo, Fred Armbrust, Caroline Schoenherr, Steffen E. Storck, Ulrich Schmitt, Silvia Zampar, Oliver Wirths, Hermann Altmeppen, Markus Glatzel, Christoph Kaether, Sascha Weggen, Christoph Becker-Pauly, Claus U. Pietrzik.

Fig S1

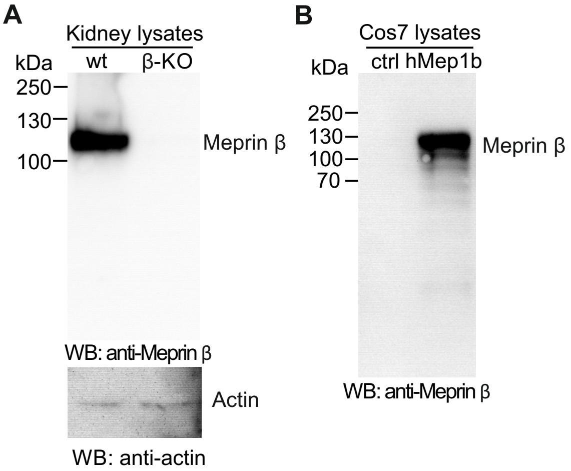

Meprin β antibody specificity analysis. (A) Isolated kidneys of representative wt and Mep1b-KO mice were lysed, equal amounts of protein per sample were loaded on SDS-PAGE and meprin β expression was analyzed by immunoblot. (B) Cos7-cells were transfected with human wt-meprin β and immunoblot analysis was performed.

Fig S2

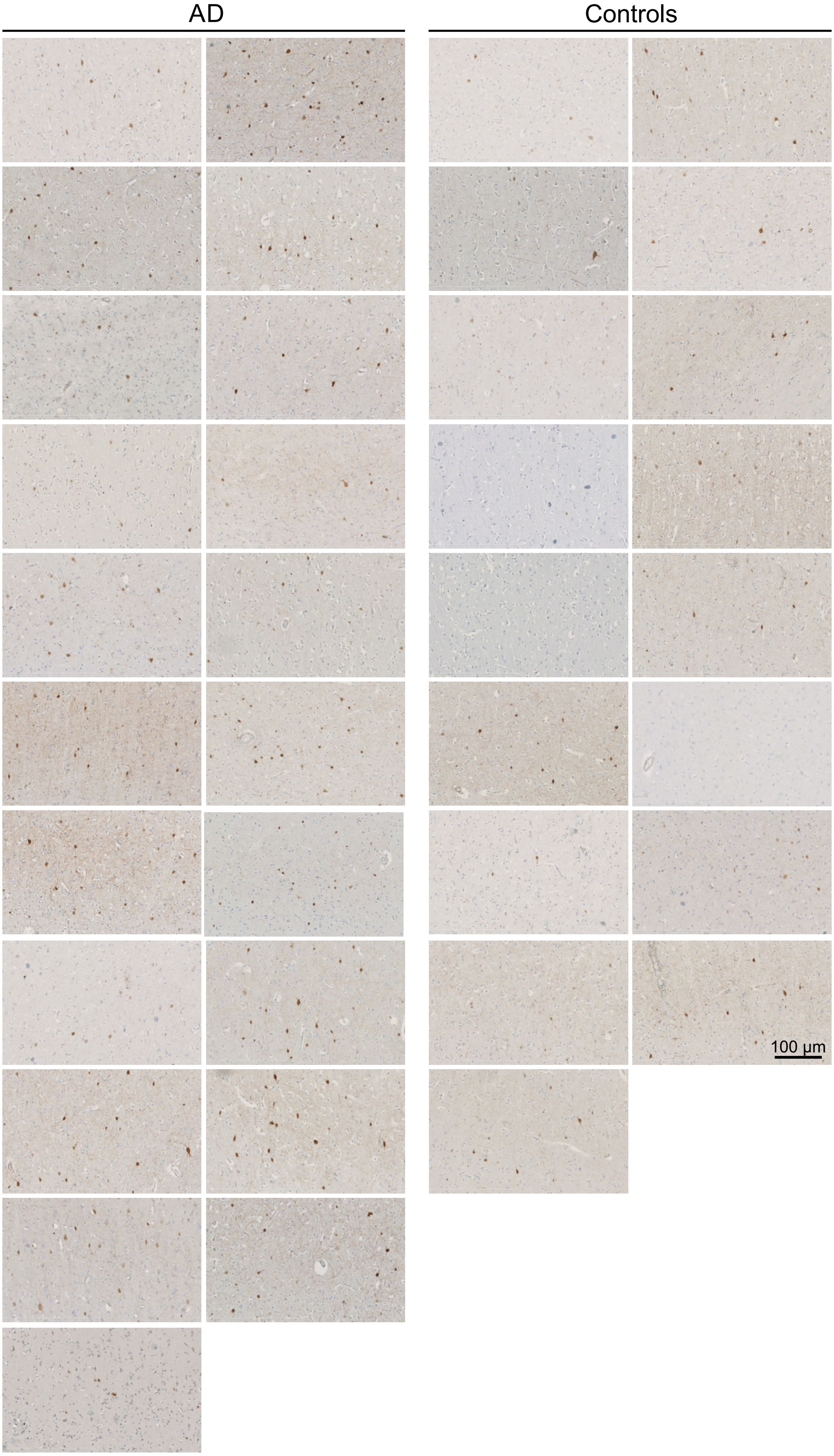

Increased meprin β expression in brains of AD patients. Immunostainings of human brain slices of AD patients, non-demented and non-AD patients were performed with polyclonal anti-meprin β antibody.

Fig S3

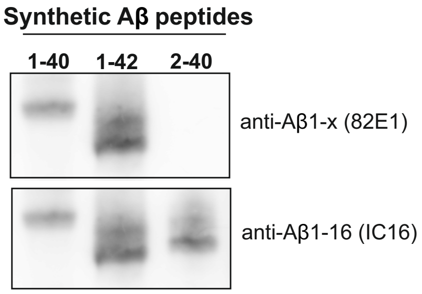

Aβ1-x antibody specificity analysis. Representative image of Urea-SDS-PAGE using synthetic Aβ peptides.The 82E1 antibody detects Aβ1-40 and Aβ1-42, but not Aβ2-40. Same membrane was reprobed with the IC16 antibody and revealed the synthetic 2-40 peptide.

Fig S4

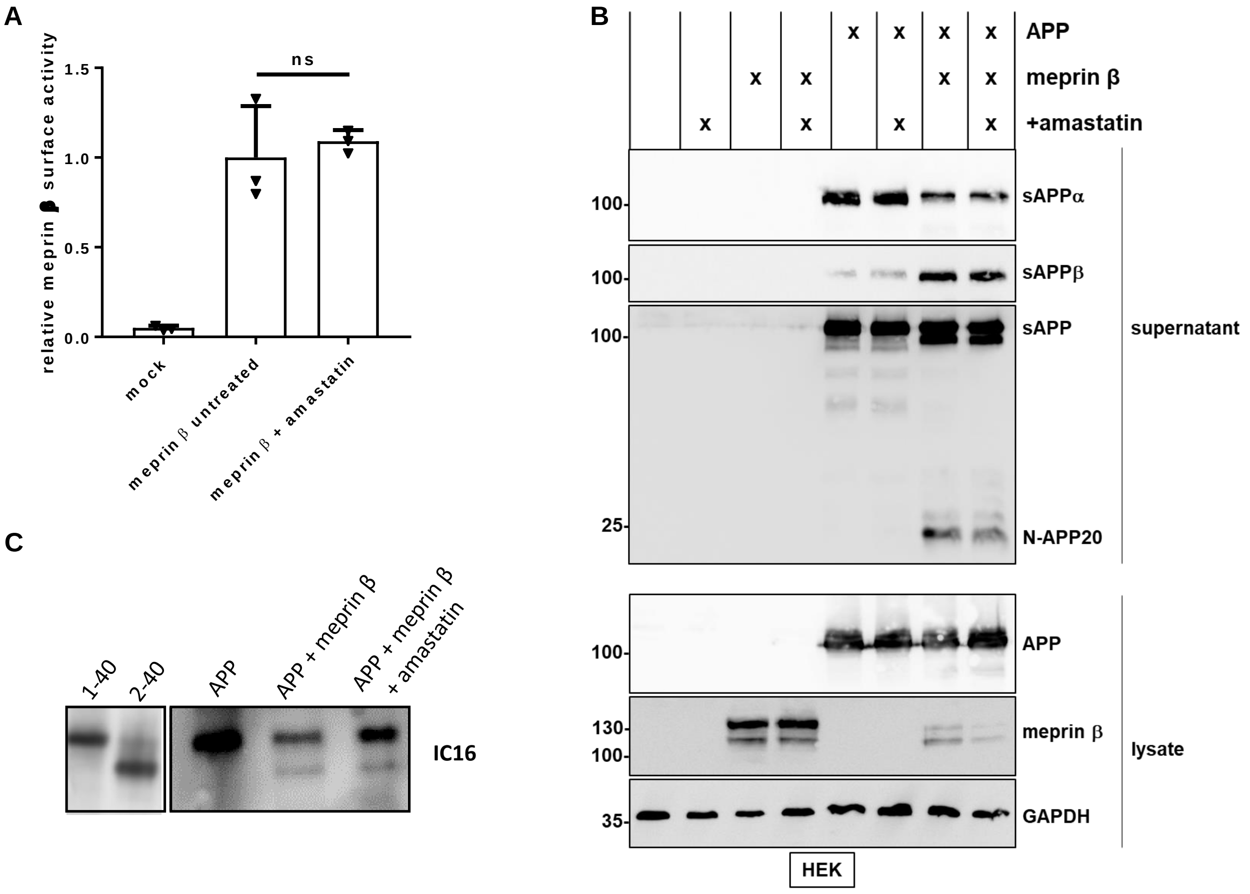

Amastatin influence on meprin β activity and Aβ production. (A) Relative meprin β surface activity showing no differences between untreated and amastatin treated cell lines (n=3 independent experiments). (B) Representative images of HEK293T cells transfected with *APP* and *Mep1b*, and treated with 10 µM amastatin. After 24 h, supernatants and lysates were analysed via SDS-PAGE. (C) Representative image of Urea-SDS-PAGE showing separation of amyloid beta peptides.

Fig S5

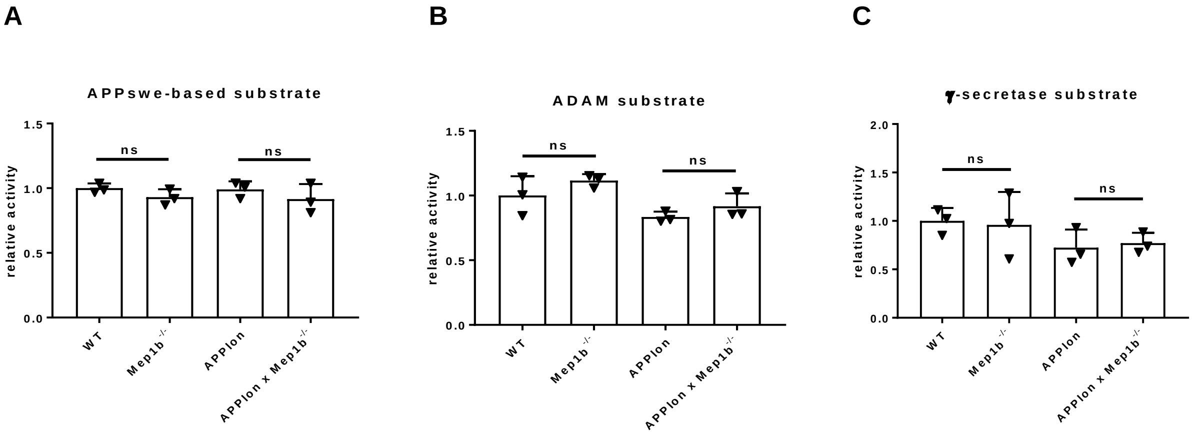

Activity assays of APP secretases. (A) An APPswe-based substrate was used as BACE1 substrate to analyse BACE1 activity related to APP. Relative activity is measured in RFU values. No differences were detected between WT and *Mep1b*$^{-/-}$ or between APP/lon and APP/lon x *Mep1b*$^{-/-}$ (n=3). (B) No differences between groups were detected for ADAM activity or (C) γ-secretase -activity.

Supplementary Material and Methods

Activity assay

In order to analyze the enzymatic activity of APP secretases in whole brain lysates, several quenched fluorescent peptide were used: ADAM substrate (dabcyl-PRYEAYKMGK-5-fam-C-NH_2, Biozyme), BACE1 substrate (abz-EVNLDAEF-N-EDdnp, Bachem), gamma-secretase-substrate (nma-GGVVIATVK-dnp-DRDRDR-NH_2, Merck). The mouse brains were lysed in 1% Triton™ X-100/PBS, pH 7.4. For measurement of ADAM substrate, EDTA-free protease inhibitor cocktail (Roche) was added; BACE-1 activity was analyzed in 1% Triton™ X-100/PBS, pH 4.5. To determine the respective secretase activity, 100 µg mouse brain lysates were analyzed. For each experiment 10 µM of the respective peptide was applied. The relative fluorescent units (RFUs) were measured with a fluorescence reader (InfiniteF200Pro or Spark™, Tecan) (emission wave lengths: 530 nm (5-fam), 420 nm (abz), 440 nm (nma); excitation wavelength: 485 nm (5-fam), 320 nm (abz), 340 nm (nma)) every 30 s for the duration of 2 h. In order to avoid the contribution of photobleaching or other unspecific alterations of the measured fluorescence, RFU values of the respective solvent mixed with 10 µM peptide substrate were determined over 2 h and subtracted from each RFU value of the measured samples. The activity values comprise the slope of the linear range, which were normalized to the wild-type values.

Cell culture and amastatin treatments

HEK293T cells were transfected with pcDNA3.1, *Mep1b* in pSG5 and *APP* isoform 695 in pCIneo using the transfection reagent polyethylenimine (PEI). Therefore, 3 µl PEI (1 mg/ml in ddH2O, Polysciences) were used per 1 µg plasmid DNA in 50 µl serum-free DMEM. 24 h after addition of the transfection mixture, the cell culture medium was exchanged with serum-free DMEM and 10 µM amastatin were added if indicated for 24 h. Cells were washed twice with PBS and subsequently lysed in 1% Triton™ X-100/PBS, pH 7.4 with protease inhibitor cocktail (Roche). The debris was removed from cell, mouse brain and OBS lysates by centrifugation for 15 min at 16,000 g and 4°C. The protein concentration of all lysates was determined using Pierce™ BCA Protein Assay Kit (Thermo Fisher Scientific) according to the manufacturer´s instructions. The samples were analyzed with SDS-PAGE and Western-Blot (Bio-Rad system) using the primary antibodies APP (22C11, 1:5000, Thermo Fisher Scientific), APP (6E10, 1:1000, Biolegend), GAPDH (14C10, 1:5000, Cell Signaling), Meprin β (Tier1, 1:1000, The antibody was generated by Pineda-Antikörper-Service against a peptide from the MAM domain of meprin β), sAPPβ (Tier3, 1:1000, The sAPPβ antibody was generated by Pineda-Antikörper-Service against the neo-C-terminus of sAPPβ, which is released after APP cleavage by meprin β between D672 and A673.

This sAPPβ form is one amino acid C-terminally extended compared to sAPPβ generated by BACE1). Secondary antibodies were purchased from Jackson Immuno Research and the signal detection was conducted with the LAS-3000 mini system (Fujifilm) using SuperSignal™ West Femto Maximum Sensitivity Substrate (Thermo Fisher Scientific) and SuperSignal™ West Pico PLUS Chemiluminescent Substrate (Thermo Fisher Scientific).

Transfections and Urea-SDS-PAGE

For analysis of amyloid beta peptides, HEK293T cells were transfected with FuGENE® HD transfection reagent (Promega, Walldorf, Germany) and treated with 100µM Amastatin (ab142479 ABCAM). After 48h, supernatants were collected and immunoprecipitated with magnetic dynabeads (M-280, 11201D, Invitrogen) coupled with 6E10 (#SIG-39320, BioLegend) according to manufacturer protocol. Urea-SDS-PAGE was performed as previously mentioned in Material and Methods section, and IC16 antibody was used for overnight immunostaining.
